# Work productivity, associated risk factors and costs on presenteeism and absenteeism in Chinese patients with young‐onset type‐2 diabetes in Hong Kong

**DOI:** 10.1111/dom.70352

**Published:** 2025-12-16

**Authors:** Juliana N. M. Lui, Kelly T. C. Wong, Eric S. H. Lau, Sunny C. S. Chan, Nga Sze Wong, Jenny Y. Z. Zhang, Kit Ming Wai, Chun Kwan O, Baoqi Fan, Hongjiang Wu, Ronald C. W. Ma, Alice P. S. Kong, Andrea O. Y. Luk, Elaine Y. K. Chow, Juliana C. N. Chan

**Affiliations:** ^1^ Department of Medicine and Therapeutics The Chinese University of Hong Kong, Prince of Wales Hospital Hong Kong SAR China; ^2^ Hong Kong Institute of Diabetes and Obesity The Chinese University of Hong Kong, Prince of Wales Hospital Hong Kong SAR China; ^3^ National Center for Mental Health China National Health Development Research Centre Beijing China; ^4^ Department of Infectious Diseases and Public Health, Jockey Club College of Veterinary Medicine and Life Sciences City University of Hong Kong Hong Kong SAR China; ^5^ Institute of Global Governance and Innovation for a Shared Future City University of Hong Kong Hong Kong SAR China; ^6^ Phase 1 Clinical Trial Centre The Chinese University of Hong Kong, Prince of Wales Hospital Hong Kong SAR China

**Keywords:** work productivity, young‐onset diabetes

## Abstract

**Aims:**

Those with young‐onset type‐2 diabetes (YOD), diagnosed before age of 40 years, experience heightened risk of complications. The economic burden extends beyond medical costs, impacting work productivity.

**Materials and Methods:**

Chinese patients with YOD were recruited between June 2023 and April 2024 in the Precision Medicine to redefine Insulin Secretion and Monogenic diabetes Trial in Hong Kong (NCT04049149). Presenteeism and absenteeism were measured using the World Health Organization Health and Performance Questionnaire.

**Results:**

Of the 639 invited participants, 603 (94%) completed the questionnaire, with 444 employed participants with type‐2 diabetes (40.1% female, 80.4% 40–50 years, 32.9% 5–10 years with diabetes, 53.3% hemoglobin A1c (HbA1c) <7.0%, 75.2% low‐density lipoprotein cholesterol <2.6 mmol/L, 42.3% body mass index ≥25 < 30 kg/m^2^). Participants reported mean presenteeism score of 7.34/10, 93.9% experiencing productivity loss with 0.48 mean sick days. Increased productivity was associated with females, child care, flexible work schedules and higher salary. Reduced productivity was related to, albuminuria, use of lipid‐lowering medications and more sick leaves. With approximately 38 700 patients with YOD in Hong Kong, territory‐wide productivity losses are projected to reach US$444 million annually (presenteeism: US$419 million, absenteeism: US$25 million).

**Conclusions:**

This study is the first to quantify productivity costs in Chinese patients with YOD, highlighting the need for work place policies, intensive treatment and management strategies to enhance support for individuals with YOD.

## 1 INTRODUCTION

Diabetes is one of the most expensive chronic conditions globally. Its rising prevalence poses a significant economic burden and challenges for healthcare systems worldwide.^1^ There are 589 million adults affected by diabetes worldwide, with global diabetes‐related health expenditures surpassing one trillion US dollars in 2024.^2^ China has the largest diabetes population with an estimated 148 million affected individuals.^2^ Asians are genetically and phenotypically more prone to develop diabetes early than non‐Asians, in part due to lower beta‐cell function and increased visceral adiposity with insulin resistance.^1^

Young‐onset type‐2 diabetes (YOD), diagnosed before the age of 40 years, is more prevalent in young to middle‐aged Asians than their Europid counterparts.^3^ Compared to those with late‐onset diabetes, patients with YOD had a two to six times higher risk of cardiovascular‐renal complications, infections and hyper/hypoglycaemia.^4^, ^5^ With the increasing prevalence of YOD and declining mortality in people with diabetes,^6^ the healthcare burden of diabetes will increase over time with huge societal implications.

Due to their working age, the financial burden of YOD extends beyond direct costs, such as medical expenses and hospitalisations.^7^, ^8^ Once diagnosed, people with YOD often need life‐long medical, psychological and behavioural care in order to maintain their well‐being and work productivity.^9^, ^10^, ^11^ Amongst patients with type‐2 diabetes (T2D) from the United Kingdom (UK), risk factors associated with reduced work productivity included poor mental health, stress, anxiety, depression, poor quality of life and history of neuropathy.^12^ In another UK nationwide cost‐of‐illness study, the estimated indirect costs related to diabetes due to absenteeism and premature mortality reached £3.28 billion in 2021–2022, more than one‐third of the estimated direct healthcare costs of £10.65 billion.^8^ According to six large employers in the United States, diabetes was ranked amongst the top 10 health conditions contributing to presenteeism costs.^13^

Diabetes is often considered a gateway to multiple traditional and non‐traditional comorbidities, increasing the risk of infections and cardiovascular–renal diseases that can affect work attendance.^14^ By assessing both overall presenteeism and absenteeism, we aim to understand the impact of diabetes and its related comorbidities on overall productivity and the implications for healthcare policies and workplace support systems.

Data on risk factors of presenteeism and related productivity loss came largely from Western countries.^15^, ^16^, ^17^, ^18^ In macroeconomic analysis using productivity‐adjusted life years (PALYs), productivity losses due to diabetes in 2018 amounted to US$2.6 trillion lost in gross domestic product (GDP) in China.^19^ In Japan, the odds of high presenteeism loss (scoring >51 out of 100) were higher in employees with diabetes (adjusted odds ratio [aOR] 1.31, 95% CI: 1.04–1.66) than their peers without diabetes, with higher odds in those receiving oral glucose‐lowering treatment (aOR 1.42, 95% confidence interval [CI]: 1.06–1.90).^20^ To date, no studies have quantified productivity loss due to presenteeism in YOD and associated risk factors.

Productivity loss studies mainly rely on validated self‐reported measures utilising composite Likert scales measuring presenteeism‐related productivity, such as the Work Productivity and Activity Impairment (WPAI) scale, Stanford Presenteeism Scale (SPS‐6) and Work Limitations Questionnaire (WLQ), Perceived Ability to Work Scale (PAWs) and the World Health Organization Health and Performance Questionnaire (WHO‐HPQ). Our team has previously conducted a thorough systematic review on productivity measures^16^ and selected the WHO‐HPQ in measuring nurse presenteeism and productivity in Hong Kong acute care hospitals.^21^ In this study, we aim to extend the WHO‐HPQ measure to patients with YOD.

This study aims to quantify presenteeism and absenteeism amongst Chinese patients with YOD and their relationships with clinical, demographic and work‐related characteristics. We further projected the potential economic impacts of productivity loss from a territory‐wide perspective to inform employers, policymakers and healthcare providers on the need to develop strategies to improve the health, well‐being and productivity of patients with YOD.

## 2 METHODS

### 2.1 Study population and recruitment

We invited Chinese patients with YOD from the Precision Medicine to Redefine Insulin Secretion and Monogenic Diabetes (PRISM) Trial in Hong Kong (NCT04049149)^22^ to participate in the Economic Impact of Diabetes and related Complications substudy (EIDC) (approval date for EIDC approval substudy: 30 May 2023). Participants were asked to complete a questionnaire after face‐to‐face informed consent during their Year 3 follow‐up visit from June 2023 to April 2024 at the Diabetes and Endocrine Research Centre at the Prince of Wales Hospital (PWH), the teaching hospital of the Chinese University of Hong Kong (CUHK). The PWH operates under the Hospital Authority, which oversees all public hospitals and clinics in Hong Kong, providing 95% of inpatient services and 80% of outpatient services across the territory.

Inclusion criteria for the PRISM trial included Chinese ethnicity, age of 18–50 years, physician‐diagnosed diabetes aged 40 years or below.^22^ Exclusion criteria included physician diagnosis of type 1 diabetes (unprovoked acute ketosis or continuous insulin requirement within 12 months of diagnosis), reduced life expectancy due to terminal illness or conditions deemed inappropriate by investigators. The study design and baseline results had been reported. In brief, 884 Chinese patients with T2D aged less than 50 and diagnosed before the age of 40 were randomised to receive usual care or multicomponent intervention delivered by a multidisciplinary team in a diabetes research centre. All patients had measurement of biogenetic markers including autoantibodies, C peptide, rare variants for 34 genes for monogenic diabetes and genetic risk scores of common variants for diabetes and its complications. The information was revealed to the intervention group with explanation by investigators for treatment adjustment with support from nurses. After 1 year of specialist care, the intervention group returned to the usual clinic with annual consultation by the specialist team at the centre. All patients underwent structured assessment at Years 0 and 3 with measurement of cardiometabolic risk factors at Years 1 and 2 at the diabetes research centre. Further details on trial design are published elsewhere.^22^ As part of the PRISM trial substudy, our reported study in this manuscript adheres to the same inclusion criteria established in the PRISM trial.

### 2.2 Assessment of presenteeism

Presenteeism was assessed using the WHO‐HPQ,^23^ a widely recognised self‐reported instrument designed to estimate the workplace costs of health problems in terms of reduced job performance and sickness absence. The WHO‐HPQ includes questions assessing both absenteeism and presenteeism. The primary outcome is presenteeism, measured by the item ‘How would you rate your overall job performance on the days you worked during the past 4 weeks (28 days)? using a visual analogue scale ranging from 0 (worst) to 10 (best).

In the regression analysis for risk factors for presenteeism, we adjusted for absenteeism estimated by the number of days of sick leave using two questions: ‘Did you miss an entire workday because of problems with your physical or mental health?’ and ‘Did you miss part of a workday due to similar issues?’ in the preceding 4 weeks.

### 2.3 Assessment of other covariates

All patients underwent structured assessment with documentation of demographics, work‐related and lifestyle factors. Demographics included age, sex, duration of diabetes, highest level of education completed, annual salary range and child care (under 18 years). Work‐related factors included employment status, work schedule (full time, part time and shift work) and annual salary. Lifestyle factors included smoking, alcohol use, frequency of exercise and adherence to a balanced diet in the past 3 months. Clinical characteristics included history of complications (coronary heart disease [CHD], peripheral vascular disease [PVD], stroke, heart failure hospitalisation, chronic kidney disease [CKD] and all site cancer), blood pressure and body mass index (BMI). Biochemical values included glycated haemoglobin, fasting plasma glucose, low‐density lipoprotein cholesterol (LDL‐C), high‐density lipoprotein cholesterol (HDL‐C), triglycerides (TG), estimated glomerular filtration rate (eGFR) and urine albumin‐to‐creatinine ratio (UACR). Use of glucose, blood pressure and lipid‐lowering drugs, including insulin were documented (Table S1).

### 2.4 Estimation of productivity loss

Costs of annual productivity loss due to presenteeism were estimated using the human capital method (HCM),^24^ We multiplied the percentage of productivity loss (e.g., self‐rated presenteeism 7 out of 10, productivity loss: (10 – 7)/10 × 100% = 30%) by the mid‐point of self‐reported annual salary range (e.g., US$25 500 was used for annual salary range US$22 000–US$29 000). The annual productivity loss costs due to absenteeism (self‐reported for the last 4 weeks) were projected by multiplying the number of days absent in the past 12 months by the mid‐point of self‐reported annual salary range.

We divided the monthly median salary range by 28 days, multiplied it by the number of working days, and applied the percentage productivity loss. Monthly productivity loss was expressed as percentage scores and converted to financial costs in US dollars, with the conversion rate set at 1 US$= 7.8 Hong Kong dollars. The reference year of salary data costs was 2023–2024 during the year of survey completion.

To project productivity loss costs of presenteeism and absenteeism to territory‐wide patients with YOD, we multiply the average annual presenteeism productivity loss and absenteeism costs per person by the number of estimated territory‐wide patients with YOD.

### 2.5 Statistical analysis

Descriptive statistics were used to summarise the demographic, clinical and work‐related characteristics of the study cohort, further stratified by sex. Continuous variables were expressed as mean/standard deviations (SD) or median, while categorical variables were presented as frequencies and percentages. The scoring conversion for presenteeism and productivity loss is based on the WHO‐HPQ guidelines.^23^ Patient characteristics between included and excluded participants were compared to evaluate any significant differences that may indicate selection bias. We used multivariable regression models to evaluate the association between presenteeism and covariates of interest expressed as beta‐coefficient (95% CI). Stepwise covariate selection was conducted to retain variables with *p* < 0.05. Based on prior knowledge, age, sex, diabetes duration and medications were fixed covariates with the inclusion of other significant covariates into the final model. Model fit was evaluated using the Akaike Information Criterion (AIC), and the final model included only those significant predictors that meaningfully contributed to the outcome. All analyses were performed using R version 4.3.2.

### 2.6 Ethical considerations

The study was approved by the Joint CUHK‐NTEC Clinical Research Ethics Committee (CREC:2019‐080‐T) with all participants giving written informed consent. Data were anonymised to protect participant confidentiality. The study was conducted in accordance with the ethical principles outlined in the Declaration of Helsinki.

## 3 RESULTS

During the follow‐up period of the PRISM Cohort at the diabetes research centre, 639 patients were invited to participate in this substudy. Of these, 589 (*n* = 92%) patients agreed to take part and completed the questionnaire. This analysis focused on 465 patients with T2D who were actively employed (Figure S1). After excluding 21 individuals with missing or inconsistent data, the final analysis included 444 patients. There are no significant differences in the majority of patient characteristics between included and excluded participants (Table S2).

At baseline, 40.1% of participants were females, 80.4% were 40‐ to 50‐year‐old, 85.1% had diabetes for 5 years or more and 39.2% held a college degree or higher (Table 1). One in three patients was caring for children under the age of 18. The majority were overweight (42.3%) or obese (30.2%). Overall, half had HbA1c above 7.0%, 24.8% had LDL‐C above 2.6 mmol/L. One in 10 had albuminuria (UACR ≥3 mg/mmol or ≥30 mg/g) and one in four had reduced kidney function (eGFR <90 mL/min/1.73 m^2^). Medication usage was high for glucose (96.4%), blood pressure (78.6%) and lipid‐lowering drugs (82.0%) with one in four treated with insulin (25.5%). For complications, 6.1% had CKD (eGFR<60 mL/min/1.73 m^2^), 4.8% had CHD and 3.4% had any‐site cancer at baseline. Most (94.4%) patients reported adherence to a balanced diet in the past 3 months with 68% having at least 150 min of regular exercise weekly. One in five were current smokers (20.7%) and 43.3% occasionally or regularly drank alcohol. Most of them were full‐time employees (82.4%) with regular working hours (81.8%) and 38% had an annual salary range of US$29 000–45 000 (Table 1).

**TABLE 1:** Baseline characteristics of participants with young‐onset type 2 diabetes diagnosed under the age of 40 years.

Variables (*n* = 444)	*n* (%)	
Personal characteristics		
Female	177	(40.1)
Age		
≥40 < 50 years old	357	(80.4)
≥50 years old	87	(19.6)
Duration of diabetes		
<5 years	66	(14.9)
≥5 < 10 years	146	(32.9)
≥10 < 15 years	128	(28.8)
≥15 years or more	104	(23.4)
Education		
College or above	174	(39.2)
High school	113	(35.4)
Middle, primary school or lower	157	(25.5)
Taking care of children <18 years		
Yes	147	(33.1)
Clinical characteristics		
HbA1c (%)		
<7.0	236	(53.2)
≥7.0	208	(46.8)
Low‐density lipoprotein cholesterol (mmol/L)		
<2.60	334	(75.2)
≥2.60 < 3.35	76	(17.1)
≥3.35	34	(7.7)
Albuminuria status		
Yes (UACR ≥3 mg/mmol/≥30 mg/g)	47	(10.6)
Estimated glomerular filtration rate (eGFR) (mL/min/1.73 m^2^)		
<60	27	(6.1)
≥60 < 90	88	(20.0)
≥90	326	(73.9)
Body mass index (kg/m^2^)		
<25	122	(27.5)
≥25 < 30	188	(42.3)
≥30	134	(30.2)
Medications		
Insulin	113	(25.5)
Lipid‐lowering drugs	364	(82.0)
Blood pressure lowering drugs	349	(78.6)
Oral glucose‐lowering drugs	428	(96.4)
History of complications		
Coronary heart disease	21	(4.8)
Peripheral vascular disease	3	(0.7)
Stroke	7	(1.6)
Heart failure hospitalisation	2	(0.5)
Chronic kidney disease	27	(6.1)
Any‐site cancer	15	(3.4)
Lifestyle factor		
Adherence to balanced diet in past 3 months	419	(94.4)
Frequency of exercise per week		
No regular activity	141	(32.0)
Less than three times	175	(39.7)
Three to four times	43	(9.8)
Five times or more	82	(18.6)
Smoking		
Current	92	(20.7)
Ex‐smoker	57	(12.8)
Never	295	(66.4)
Use of alcohol		
Regular	14	(3.2)
Occasional	178	(40.1)
Ex‐drinker	19	(4.3)
Never	233	(52.5)
Work factors		
Employment status		
Full time	366	(82.4)
Part time	24	(5.4)
Self‐employed	54	(12.2)
Work schedule		
Regular working hours	363	(81.8)
Shift work/self‐employment	81	(18.2)
Annual salary (US$)		
<22 000	45	(10.1)
≥22 000 < 29 000	69	(15.5)
≥29 000 < 45 000	171	(38.5)
≥45 000 < 71 000	95	(21.4)
≥71 000	64	(14.4)

*Note*: Data are *n* (%) or mean (standard deviation). Glucose‐lowering drugs included alpha‐glucosidase inhibitor (AGIs), dipeptidyl peptidase 4 (DPP‐4) inhibitor, meglitinide, metformin, sodium‐glucose cotransporter‐2 (SGLT2) inhibitor, sulfonylurea and thiazolidinedione.

Abbreviation: UACR, urine albumin‐to‐creatinine ratio.

### 3.1 Presenteeism and absenteeism in patients with YOD

Table 2 summarises self‐reported presenteeism and absenteeism during the past 28 days. The performance rating was 7.34 (SD: 1.44) out of 10 with 93.9% having experienced productivity loss (presenteeism). The average total sick leave taken was 0.48 (SD: 1.48) days with 22.1% having taken at least half a day of sick leave during the same period.

**TABLE 2:** Self‐reported presenteeism, absenteeism and productivity loss in patients with young‐ onset type 2 diabetes diagnosed under the age of 40 years.

*n* = 444	Mean (SD)/*n* (%)
Presenteeism	
Self‐rated overall performance^a^	7.34 (1.44)
Number of people who rated loss in productivity (presenteeism) in the past 28 days^b^	417 (93.92%)
Absenteeism	
Total days of sick leave^c^	0.48 (1.48)

Abbreviation: SD, standard deviation.

^a^ Self‐rated performance was assessed by the item asking participants' overall job performance on the days you worked during the past 4 weeks (28 days), rated on a scale from 0 to 10, translated into percentage.

^b^ Number of people who scored less than 10 on the item asking participants' overall job performance on the days you worked during the past 4 weeks (28 days), rated on a scale from 0 to 10.

^c^ Sick leave days were assessed by two questions about missing an entire or part of a workday due to health issues in the past 4 weeks.

### 3.2 Association of risk factors with work productivity

**FIGURE 1:**
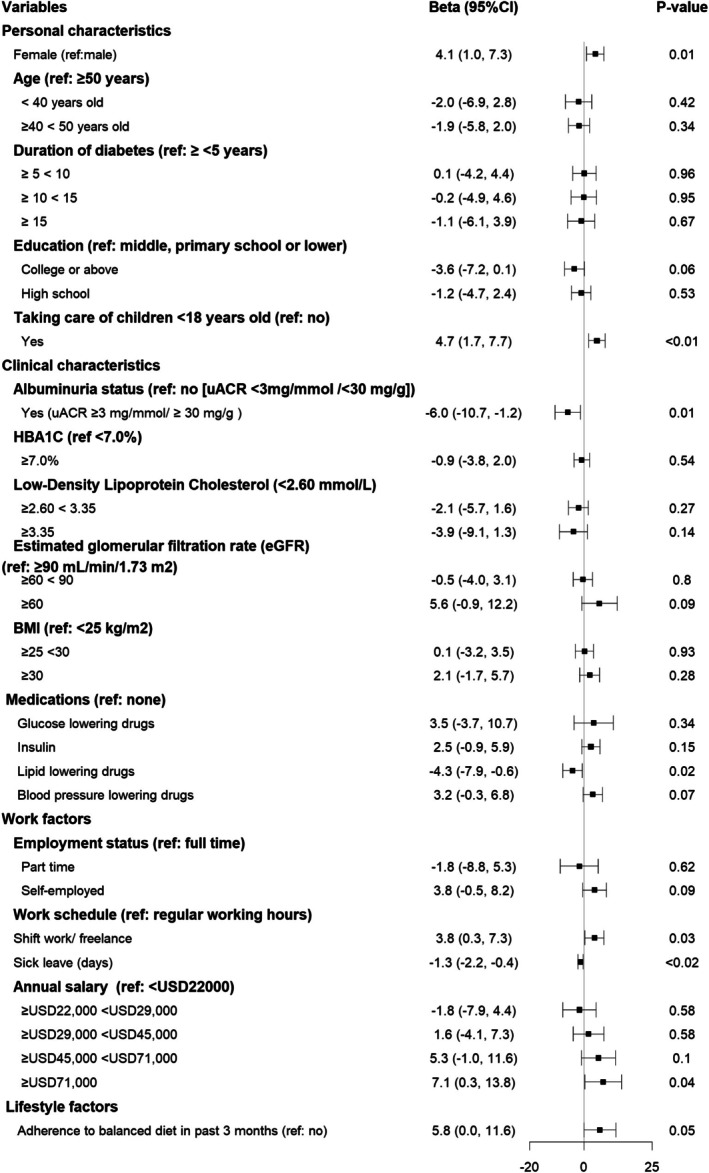
Forest plot on the associations of risk factors for work productivity in patients with young‐onset type 2 diabetes diagnosed under the age of 40 years (*n* = 444). Dependent variable in the regression analysis is self‐rated performance was assessed by the item asking participant's overall job performance on the days you worked during the past 4 weeks (28 days), rated on a scale from 0 to 10, translated into percentage. BMI, body mass index; UACR, urine albumin‐to‐creatinine ratio.

Figure 1 illustrates the risk factors associated with work productivity. Overall, work productivity, measured on a scale of 0–100, was reported to be higher in several groups: females (beta estimate: 4.1, 95% CI: 1.0, 7.3) compared to men, individuals taking care of children (4.7, 95% CI: 1.7, 7.7) relative to those without children, those working shifts or freelance had a productivity advantage (3.8, 95% CI: 0.3, 7.3) compared to those on regular work schedules; and participants earning US$71 000 or more annually exhibited higher productivity (7.1, 95% CI: 0.3, 13.8) compared to those earning less than US$22 000 annually. Conversely, work productivity was negatively associated with several health factors: albuminuria (−6.0, 95% CI: −10.7, −1.2) compared to those without this condition, lipid‐lowering drugs (−4.3, 95% CI: −7.9, −0.6) compared to those not on these medications, and reporting a higher number of leave days (−1.3, 95% CI: −2.2, −0.4) was associated with lower productivity.

### 3.3 Projected economic Impact of productivity loss in patients with YOD

The average 26.6% (SD: 14.4%) productivity loss due to presenteeism, when applied to self‐reported salary, translated to an annual cost of US$10 834 (SD: US$7254) per person (Table 3). The average of 6.3 sick leave days (SD: 19.3) per year was associated with a cost of US$647 (SD: US$1904) per person. The estimated total annual costs from absenteeism and presenteeism were US$11 481 (SD: US$7744) per person.

**TABLE 3:** Annual estimate of total productivity loss in patients with young‐onset type 2 diabetes diagnosed under the age of 40 years.

Estimation of indirect costs due to absenteeism and presenteeism in patients with young‐onset diabetes	Estimates	
	Mean	(SD)
Presenteeism		
Self‐rated productivity loss (%)^a^	26.6	(14.4)
Annual on‐the‐job productivity loss cost (US$/person)	10 834	(7254)
Absenteeism		
Annual number of sick leave days per person (days)^b^	6.3	(19.3)
Annual sick leave costs (US$/person)	647	(1904)
Annual total absenteeism and presenteeism costs (US$/person)	11 481	(7744)
Territory‐wide projection on presenteeism and absenteeism costs		
Number of patients with young‐onset diabetes in territory‐wide Hong Kong	38 700	
Projected annual territory‐wide productivity loss costs (US$ million)	419	(281)
Projected annual territory‐wide sick leave costs (US$ million)	25	(74)
Projected annual costs due to absenteeism and presenteeism (US$ million)	444	(298)

Abbreviation: SD, standard deviation.

^a^ (10 − self‐rated performance on a scale of 0–10) × 100%. Self‐rated performance was assessed by the item asking participants' overall job performance on the days you worked during the past 4 weeks (28 days), rated on a scale from 0 to 10, translated into percentage.

^b^ Self‐reported sick leave days over the past 4 weeks (28 days) averaged 0.48 days, translating to an annual estimate of 6.25 days when multiplied by 365 days.

Hong Kong has a population of 7.4 million with over 90% managed under the Hospital Authority. With approximately 38 700 patients with YOD,^25^ we projected the territory‐wide annual productivity loss costs due to presenteeism and absenteeism to reach US$419 million (SD: US$281 million) and US$25 million (SD: US$74 million), respectively, giving a total annual loss of US$444 million (SD: US$298 million).

## 4 DISCUSSION

To our knowledge, this is the first study that quantified the risk factors and financial burden of presenteeism and absenteeism in YOD. In this Chinese cohort of YOD, 93% reported productivity loss related to presenteeism, as defined by a score less than 10 based on self‐rated performance in WHO‐HPQ. In addition, one in five (22%) had taken sick leave in the past month. While female gender, child care, flexible working hours, high salaries and self‐employment were associated with increased productivity, suboptimal health as indicated by albuminuria and use of lipid‐lowering drugs, was associated with reduced productivity. In the territory‐wide electronic medical record (EMR: 1995–2016), there were 38 700 patients with YOD in the public healthcare system.^25^ Based on our results, we projected an annual productivity loss of US$444 million associated with YOD, of which 94% were attributed to presenteeism.

### 4.1 High prevalence of presenteeism in Asians

Our findings concurred with the high prevalence of presenteeism and suboptimal work productivity associated with chronic conditions.^13^, ^20^, ^26^, ^27^ Individuals with diabetes experienced higher productivity loss than those without diabetes.^20^, ^28^, ^29^ However, the use of different measurement tools limited direct comparison across studies.^30^ Other than the WHO‐HPQ used in our study, other common measurement tools for presenteeism or absenteeism included the WPAI,^31^ WLQ^32^ and self‐designed questions.^16^, ^33^

Compared to studies utilizing the WHO‐HPQ measure, our reported mean work productivity of 73.4% in patients with YOD was lower than 81.3% reported amongst newly diagnosed patients with diabetes (18–63 years) in the South London Diabetes (SOUL‐D).^34^ The self‐reported work performance in our cohort was also lower than that observed in a US claims analysis in patients with T2D (85.8%–86.6%) with an age range of 45–55 years old treated with statins, Angiotensin‐Converting Enzyme inhibitors inhibitors (ARB), oral glucose‐lowering drugs or insulin.^35^

Cultural factors might influence self‐reported presenteeism across different geographical regions.^36^, ^37^ A Japanese study applied the WHO‐HPQ to 9366 corporate employees and reported an overall work performance of 60.5% regardless of their disease status.^27^ This figure was considerably lower than that in predominantly Europid cohorts.^34^, ^35^ In a cross‐cultural study, self‐reported presenteeism was more prevalent amongst Chinese employees than their counterparts in the UK regardless of health status with Chinese workers reporting higher stress levels. The authors postulated that this inter‐ethnic difference might stem from Confucian cultural values in Asians which emphasised persistence and endurance with individuals tending to attend work despite feeling unwell.^37^

Our reported absenteeism of 0.48 days translated to approximately 6.3 sick days per year. This represented the lower end compared to 5.8–18.1 sick days reported in a systematic review which included studies mainly from the Europid population with diabetes.^17^ The difference across our Chinese cohort and the Europid population may reflect variations in cultural attitudes (e.g., Confucian values) mentioned earlier towards illness and work.^37^ In the same analysis, the authors reported higher mean absenteeism rates in those with diabetes (5.4–18.1 days per year) than those without diabetes (3.4–8.7 days per year). Furthermore, those with T2D and comorbid depressive symptoms reported an average of 78.5 lost workdays.^17^ By comparison, our YOD cohort reported fewer hours of absence equivalent to 3.8 h for an 8‐h workday per month, that is, 6.3 days per year. This was compared to 2.4–7.7 h per month (3.6–11.6 days per year) reported by US patients with diabetes on various treatments.^35^

### 4.2 Risk factors associated with work productivity

Our study identified a multitude of demographic, clinical and work‐related factors associated with work productivity in YOD. These attributes provided important insights on the importance of targeted clinical interventions and workplace support to improve their work performance and well‐being in high‐risk individuals. Despite having a longer duration of diabetes, women with YOD in our cohort had a lower prevalence of CKD and higher productivity than men (Table S3). Reasons underlying this gender difference were not immediately obvious since favourable factors for work productivity, such as child care, flexible working hours and higher salaries were less represented in women. Gender differences in coping mechanisms and personality warrant further investigations.

Other studies have shown that employees with a higher health locus of control who believed in one's ability to influence one's own health experienced lower presenteeism. These individuals were able to cope with job demands despite feeling unwell.^38^ This high level of control might also explain the better productivity in higher income earners (>US$71 000). In a Finnish survey, nurses with a strong locus of control and high responsibilities might have more autonomy to adjust resources at work and alleviate stressors with improved work performance.^39^ This short‐term high performance with a high‐demanding job might eventually lead to burnout and presenteeism.^40^ Interestingly, patients who had to care for dependent children had better overall work productivity. Our findings aligned with previous research highlighting family responsibilities as an important factor to motivate performance which could be enhanced by supervisors with family‐supportive behaviours.^41^

The relationship between the regularity of working hours and productivity remained inconclusive. In a Korean report, fixed night shifts were associated with health‐related productivity loss.^42^ In a Cochrane systematic review, flexible working hours were associated with positive health outcomes and well‐being.^43^ In Korea, nurses identified health problems, sleep disturbances and inadequate rest between breaks during shifts as factors that increased sick leaves and presenteeism. In our cohort, we observed higher productivity amongst shift and freelance workers than those with regular working hours.^44^ We speculate that flexible working hours might enable a better balance between work and personal life. This flexibility also posed fewer challenges in adhering to schedules of diabetes care, for example, regular follow‐up visits and assessments. In a recent survey, a hybrid mode of work (e.g., 2 days of work from home) did not negatively affect work performance. On the other hand, shift work might impose long‐term strains, particularly on those with poor health,^40^ although we did not obtain full details of shift duties or freelance work in our cohort.

### 4.3 Preventing complications to mitigate productivity loss in patients with YOD

Prevention of complications and health preservation are crucial in maintaining work productivity. In our current cohort, less than 6.5% of our patients had complications at baseline. Patients with severe hypoglycaemia had a 50% productivity loss compared to those without.^45^ Diabetes‐related neuropathic symptoms incurred US$3.65 billion per year due to health‐related productivity loss.^46^ In our study, patients with YOD treated with lipid‐lowering medications and those with albuminuria took more sick leaves which were closely associated with productivity loss. In part due to long‐term exposure to an abnormal milieu, patients with YOD had a 1.5‐ to 3‐fold higher risk of cardiovascular‐renal events and all‐cause death than their peers diagnosed after the age of 40 years.^1^

In a Korean study, early intensified treatment could prevent 1700 micro‐ and macrovascular events with an annual societal gain of US$23 million due to increased productivity.^47^ Using a territory‐wide EMR (*n* = 257 280) and productivity measures reported from literature, our team estimated that the total PALYs amongst the working population with T2D in Hong Kong resulted in a GDP loss of US$15.3 (95% CIs: 15.2, 15.4) billion in men and US$14.5 (95% CIs: 14.4, 14.6) billion in women. In particular, younger individuals (20–24 years) experienced a loss in PALYs that was 13–16 times greater than older individuals (60–64 years).^48^ Our analysis complemented these results by quantifying presenteeism and absenteeism and their risk factors. Results from this 3‐year PRISM RCT^22^ will provide insights into whether intensive treatment using a multicomponent approach would improve risk factors, reduce complications and increase productivity in YOD.

### 4.4 Economic impact of diabetes on work productivity

The economic burden of diabetes on work productivity is substantial. This is the first study to date in quantifying the annual productivity loss in patients with YOD. In the entire population with diabetes under the public healthcare system, 7% had YOD. Based on our findings, we estimated the overall presenteeism (US$419 million) and absenteeism (US$25 million) in YOD would result in an overall loss of US$444 million per year. This is equivalent to a US$1 dollar productivity loss for every US$917 of GDP in Hong Kong in 2024.^49^ In the United States, diabetes was estimated to cause a productivity loss of 112.7 million days and a cost of US$35.8 billion. The accompanying 17.3 million days of absenteeism resulted in an additional US$5.4 billion in 2022.^26^

Analysis of a Vietnam insurance claims database revealed a total direct and indirect cost of US$239 million due to diabetes in 2017. Absenteeism, presenteeism and premature mortality contributed to 17%, 73% and 10% of the indirect costs, respectively.^50^ In the Gulf Cooperation Council, which includes Kuwait, Oman, Qatar, Saudi Arabia and the United Arab Emirates, productivity loss related to T2D amounted to international dollar of Int$36.9 billion (absenteeism Int$2.4 billion, presenteeism Int$34.5 billion) which was half of the total productivity loss (Int$80.6 billion). This loss was mainly attributable to seven major noncommunicable diseases, including CHD, stroke, breast cancer, colon cancer, chronic obstructive pulmonary disease and asthma.^51^

Due to a lack of patient‐level data, most macroeconomic studies from lower‐ and middle‐income countries used PALYs as a proxy of productivity loss.^19^, ^52^ In 2017, amongst 56.4 million individuals of working age in China, 7.1% had diabetes. Researchers projected an estimated average loss of 1.3 PALYs per person with diabetes. This translated to an annual GDP loss of US$2.6 trillion due to reduced productivity, that is, US$45 959 per person per year.^19^ Together with these results on YOD, urgent actions including work policies are needed to optimise productivity and reduce absenteeism in our work force.^1^, ^53^

### 4.5 Strengths and limitations of the study

This study has multiple strengths. The participation rate was 94% which enhanced the reliability of our findings. The comprehensive risk profiles documented through structured assessment provided a broad range of covariates to explore risk factors for work productivity, presenteeism and absenteeism. These data generated hypothesis for further testing to evaluate policies and care strategies to reduce the economic burden of YOD. This randomised control trial cohort samples patients with YOD in Hong Kong visiting our ambulatory specialist clinic, with comprehensive data collected through nurse assessments with minimal missing information. The rich dataset includes demographic, clinical and patient‐reported outcomes, allowing for an in‐depth understanding of patients with YOD and providing a strong foundation for assessing its impact in Hong Kong.

However, several limitations must be considered. The reliance on self‐reported measures might introduce bias, as participants tended to under‐report absenteeism or over‐estimate productivity. Furthermore, reported estimates are self‐reported, within‐group measures and do not constitute comparisons to other populations or time points. We acknowledge that self‐reported salary data may introduce bias; however, obtaining alternative salary data, such as tax records, is not feasible in our region due to privacy concerns, making self‐reported data the most viable option for cost calculations. Study results should be interpreted with caution and apply to the local Hong Kong setting only, as diabetes‐related productivity loss is difficult to compare internationally due to differences in wages, labour structures and social insurance systems. There are limitations in indirect costing studies with ongoing methodological improvement to develop, evaluate and validate objective measures for presenteeism and productivity loss. The cross‐sectional design limits causal inferences. Despite the high participation rate, selection and volunteer bias called for study extension to include more patients from diverse healthcare settings. Our YOD cohort was recruited from hospital‐based clinics that might have a more complex clinical course than their peers in primary care settings. On the other hand, we might under‐estimate the economic impact of YOD since patients with more severe diseases were not captured. Our findings were specific to Chinese and might not generalise to other contexts. The lack of longitudinal data limited the assessment of relationships between changes in health outcomes and productivity over time.

Future research can be extended to participants from primary care and community settings and utilise longitudinal designs to track productivity changes over time to gain more insights into the economic impact of YOD. We previously reported that patients with YOD are at a heightened risk for mental health issues.^54^ Therefore, future studies may explore the association between mental health and diabetes in relation to absenteeism and overall productivity. Additionally, validation studies on various presenteeism instruments in Asian patients with YOD should be conducted, as this area remains underexplored. Furthermore, heterogeneity amongst employees across different sectors warrants further investigation into how work factors, such as job demands, workload and stress influence work performance, particularly through the lens of theoretical frameworks such as the Job Demands‐Resources model.^16^ Examining socio‐cultural and personal factors, such as personality, motivation and job flexibility, may provide insights into their roles as potential mediators in the observed relationships of this study.

## 5 CONCLUSION

For the first time, we have highlighted the impact of productivity loss through presenteeism and absenteeism in YOD. Demographics, family background, work nature and health status were independently associated with productivity in YOD. These findings emphasise the importance of family and work life on health in our workforce. Our results call for a holistic research, implementation and evaluation programme aimed at fostering a work environment that prioritises health and well‐being to transform practice and policies for the betterment of our work force.

## CONFLICT OF INTEREST STATEMENT

Juliana NM Lui has received research funding from Roche Diagnostics Hong Kong Ltd for projects that are unrelated to the work presented in this manuscript. Juliana CN Chan has received grants and/or honoraria for consultancy or lectures from Applied Therapeutics, AstraZeneca, Bayer, Boehringer Ingelheim, Celltrion, Eli Lilly, Hua Medicine, Powder Pharmaceuticals, Merck, MSD, Pfizer, Sanofi, Servier, Viatris, and Zuelig Pharma. Alice PS Kong has received honoraria for consultancy or lectures from Abbott, AstraZeneca, Bayer, Boehringer Ingelheim, Eli Lilly, Kyowa Kirin, Merck Serono, Nestlé, Novo Nordisk, Pfizer, and Sanofi. Andrea OY Luk has received research grants, honoraria, and/or served on advisory panels for Amgen, AstraZeneca, Bayer, Boehringer Ingelheim, Lee’s Pharmaceutical, MSD, Novo Nordisk, Roche, Sanofi, Sugardown Ltd, and Takeda, outside the submitted work. Ronald CW Ma has received research grants and/or honoraria for consultancy or lectures from AstraZeneca, Boehringer Ingelheim, Bayer, Kyowa Kirin, Merck, Novo Nordisk, Pfizer, Roche Diagnostics, and Tricida Inc.; all proceeds were donated to the Chinese University of Hong Kong, the American Diabetes Association, and other charitable organizations to support diabetes research and education. All other authors declare no competing interests. Elaine YK Chow has received speaker honoraria/institutional research support from Astra Zeneca, Boehringer Ingelheim, Bayer, Merck KGaA and Sanofi. All proceeds have been donated to CUHK to support diabetes research. Other authors declare no conflicts of interest.

## Supporting information


**Data S1.** Supporting Information.

## Data Availability

The data underlying the results presented in the study are hosted by the Hong Kong Hospital Authority. Due to local regulation, the data are not available to the public. Requests for data can be made via Hong Kong Hospital Authority: https://www3.ha.org.hk/data.
