# Supplementary material for: Work productivity, associated risk factors and costs on presenteeism and absenteeism in Chinese patients with young‐onset type‐2 diabetes in Hong Kong
Source: Diabetes Obes Metab. 2025 Dec 16;28(3):1730–40. doi: 10.1111/dom.70352 (PMC12890741; doi:10.1111/dom.70352)
Supplement: Supplementary file 1 — Data S1. Supporting Information. [file DOM-28-1730-s001.docx]

**Work productivity, associated risk factors and costs on presenteeism and absenteeism in Chinese patients with young‐onset type‐2 diabetes
in Hong Kong**

Juliana NM LUI*^1,2^, Kelly TC WONG^1^, Eric SH LAU^1^, Sunny CS CHAN^1^, Nga Sze WONG^1^, Jenny YZ ZHANG^4^, Kit Ming WAI^5,6^, Chun-Kwan O^1^, Baoqi FAN^1^, Hongjiang WU^1^, Ronald CW MA^1,2^, Alice PS KONG^1,2^, Andrea OY LUK^1,2,3^, Elaine YK CHOW^1,2,3^, Juliana CN CHAN*^1,2^

^1^Department of Medicine and Therapeutics, The Chinese University of Hong Kong, Prince of Wales Hospital, Hong Kong SAR, China

^2^Hong Kong Institute of Diabetes and Obesity, The Chinese University of Hong Kong, Prince of Wales Hospital, Hong Kong SAR, China

^3^Phase 1 Clinical Trial Centre, The Chinese University of Hong Kong, Prince of Wales Hospital, Hong Kong SAR, China

^4^China National Health Development Research Centre, Beijing, China.

^5^Department of Infectious Diseases and Public Health, Jockey Club College of Veterinary Medicine and Life Sciences, City University of Hong Kong, Hong Kong SAR, China.

^6^Institute of Global Governance and Innovation for a Shared Future, City University of Hong Kong, Hong Kong SAR, China.

*co-corresponding authors

**Supplemental Material**

**Table of Contents**

[Supplementary table 1. Definitions of covariates included in the study 2](#_Toc198804345)

[Supplementary table 2. Participant inclusion flowchart 4](#_Toc198804346)

[Supplementary table 3. Patient characteristics table stratified by sex 5](#_Toc198804347)

# Supplementary table 1. Definitions of covariates included in the study

| **Covariates** | **Categories (if available)** | **Remarks** |  |
| --- | --- | --- | --- |
| **Demographics and lifestyle** | | | |
| Sex | Male  Female |  |  |
| Age (years-old) | < 40  40-59  ≥ 60 |  |  |
| Age at diagnosis  (years-old) | < 40  40-59  ≥ 60 |  |  |
| Number of years with diabetes (years) | <5  5 - 9  10 - 14  ≥ 15 |  |  |
| Education level | Primary, illiterate or others  Middle or high school  College or above |  |  |
| **Clinical and biochemical characteristics** | | | |
| Body mass index (BMI) (kg/m^2^) | <25  25 - 30  ≥ 30 or more | We followed the BMI cut-off as stated by the World Health Organization (WHO) for Asians (WHO Expert Consultation, 2004 The Lancet, 363(9403), 157–163) |  |
| HbA1c (%) | < 7.0  ≥ 7.0 |  |  |
| LDL-cholesterol (mmol/L) | <2.6  ≥2.6 < 3.35  ≥3.35 |  |  |
| Estimated glomerular filtration rate (ml/min/1.73m^2^) | < 90  ≥ 90 | Calculated using the CKD-EPI formula |  |
| Albuminuria status | Yes  No | Albuminuria is defined as uACR ≥3 mg/mmol or  ≥30 mg/g |  |
| **Medical treatments** | | | |
| Oral glucose-lowering drugs |  | Alpha-glucosidase inhibitor (AGIs), dipeptidyl peptidase 4 (DPP-4) inhibitor, meglitinide, metformin, sodium-glucose cotransporter-2 (SGLT2) inhibitor, sulphonylurea, thiazolidinedione |  |
| Insulin |  |  |  |
| Lipid lowering drugs |  | Ezetimibe, cholestyramine, fibrate, statins |  |
| Blood-pressure lowering drugs |  | Alpha blocker, beta blocker, calcium channel blocker, hydralazine, methyldopa, nitrate, thiazide, Angiotensin-converting enzyme (ACE) inhibitor, Angiotensin receptor blocker (ARB) |  |
| **History of complications** | | | |
| Coronary heart disease | Yes  No | 1. Acute myocardial infarction (410) 2. Other acute and subacute forms of ischemic heart disease (411) 3. Old myocardial infarction (412) 4. Angina pectoris (413) 5. Other forms of chronic ischemic heart disease (414) |  |
| Peripheral vascular disease | Yes  No | 1. Diabetes with peripheral circulatory disorders (250.7) 2. Gangrene (785.4) 3. Peripheral angiopathy in diseases classified elsewhere (443.81) 4. Peripheral vascular disease, unspecified (443.9) 5. Procedures: Other (peripheral) vascular shunt or bypass (procedure code 39.29); insertion of non-drug-eluting peripheral vessel stent(s) (39.90); 38.08, 38.18, 38.38, 38.48, 38.68, 38.88, 39.25, 39.49, 39.56, 39.57, 39.58, 39.59, 39.99; 00.55, 17.56, 39.50, 39.79 |  |
| Stroke | Yes  No | 1. Occlusion and stenosis of precerebral arteries (433) 2. Occlusion of cerebral arteries (434) 3. Subarachnoid hemorrhage (430) 4. Intracerebral hemorrhage (431) 5. Other and unspecified intracranial hemorrhage (432) |  |
| Heart failure hospitalization | Yes  No | 1. Heart failure (428) |  |
| Chronic kidney disease | Yes  No | Defined according to the Kidney Disease: Improving Global Outcomes (KDIGO) guidelines, CKD was classified based on estimated glomerular filtration rate (eGFR) category into 5 stages (G1-G5). CKD G3 and 4 (GFR 59-15 ml/min/1.73m2) represent loss of 50% or more of normal kidney function and are seen as a cut-off for clinically significant CKD, whereas G5 covers GFR under 15 ml/min/1.73m2 ^1^. |  |
| All-site cancer | Yes  No | Neoplasms (140-208) |  |
| **Lifestyle characteristics** | | | |
| Taking care of children | Yes  No | Children < 18 years |  |
| Adherence to balanced diet in past 3 months | Yes  No |  |  |
| Regular physical activity in last 3 months | No regular activity  Less than 3 times  3 to 4 times  5 times or more | At least 150 minutes per session |  |
| Smoking | Current  Ex-smoker  Never |  |  |
| Use of alcohol | Regular  Occasional  Ex-drinker  Never |  |  |
| **Work characteristics** | | | |
| Employment | Full time  Part time  Self-employed |  |  |
| Work schedule | Regular working hours /  Shift-work / freelance | Regular working hours = 9am-5pm |  |
| Annual salary (USD $) | <22,000  ≥22,000 <29,000  ≥29,000 <45,000  ≥45,000 <71,000  ≥71,000 |  |  |

1. Rossing P, Caramori ML, Chan JC, Heerspink HJ, Hurst C, Khunti K, Liew A, Michos ED, Navaneethan SD, Olowu WA. KDIGO 2022 clinical practice guideline for diabetes management in chronic kidney disease. Kidney international. 2022;102(5):S1-S127.

# Supplementary figure 1. Participant inclusion flowchart

884 patients

Excluded patients who did not receive face-to-face informed consent for this study during follow-up visit = 245

Refuse to participate/did not complete questionnaire = 50

Missing data for items on presenteeism and absenteeism = 8

Extreme outliers on sick days = 1

Type 1 diabetes = 5

Maturity onset diabetes of the young = 16

Unemployed, temporarily laid off, retired or full-time student =103

Multiple/conflicting employment status = 2

Missing data on key variables (e.g. salary range for cost analysis) =10

444 patients

453 patients

465 patients

589 patients

639 patients

# Supplementary table 2. Patient characteristics of included and excluded participants

|  | **Included participants**  (n=444)  N (%) /  Mean (SD) | | **Excluded participants**  (n=145)  N (%) /  Mean (SD) | | **P-value** |
| --- | --- | --- | --- | --- | --- |
|  |  |  |  |  |  |
|  |  |  |  |  |  |
| ***Personal Characteristics*** |  |  |  |  |  |
| ***Sex*** |  |  |  |  |  |
| Male | 266 | (59.9) | 64 | (44.1) | 0.001 |
| Female | 178 | (40.1) | 81 | (55.9) |  |
| **Education** |  |  |  |  |  |
| College or above | 174 | (39.2) | 56 | (38.6) | 0.941 |
| High school | 113 | (25.5) | 39 | (26.9) |  |
| Middle, primary school or lower | 157 | (35.4) | 50 | (34.5) |  |
| **Age** |  |  |  |  |  |
| < 40 years old | 106 | (23.9) | 44 | (30.3) | 0.131 |
| ≥ 40 < 50 years old | 251 | (56.5) | 75 | (51.7) |  |
| ≥ 50 years old | 87 | (19.6) | 25 | (17.2) |  |
| Missing | 0 | (0.0) | 1 | (0.7) |  |
| **Duration of diabetes** |  |  |  |  |  |
| <5 years | 66 | (14.9) | 24 | (16.6) | 0.464 |
| ≥ 5 < 10 years old | 146 | (32.9) | 51 | (35.2) |  |
| ≥ 10 < 15 years old | 128 | (28.8) | 32 | (22.1) |  |
| ≥ 15 years old | 104 | (23.4) | 38 | (26.2) |  |
| ***Clinical characteristics*** |  |  |  |  |  |
| **HbA1c (%)** |  |  |  |  |  |
| <7.0 | 236 | (53.2) | 71 | (49.0) | 0.435 |
| ≥7.0 | 208 | (46.8) | 74 | (51.0) |  |
| **Low-Density Lipoprotein Cholesterol (mmol/L)** |  |  |  |  |  |
| <2.60 | 334 | (75.2) | 115 | (79.3) | 0.051 |
| ≥2.60 <3.35 | 76 | (17.1) | 19 | (13.1) |  |
| ≥3.35 | 34 | (7.7) | 9 | (6.2) |  |
| Missing | 0 | (0.0) | 2 | (1.4) |  |
| **Albuminuria status** |  |  |  |  |  |
| Yes (uACR ≥3 mg/mmol / ≥30 mg/g) | 47 | (10.6) | 13 | (9.0) | 0.688 |
| **Estimated glomerular filtration rate (eGFR) (mL/min/1.73m^2^)** |  |  |  |  |  |
| <60 | 17 | (6.4) | 10 | (5.6) | 0.002 |
| ≥60 <90 | 67 | (25.4) | 21 | (11.9) |  |
| ≥90 | 180 | (68.2) | 146 | (82.5) |  |
| **Body Mass Index (kg/m^2^)** |  |  |  |  |  |
| <25 | 122 | (27.5) | 58 | (40.0) | 0.017 |
| ≥25 <30 | 188 | (42.3) | 49 | (33.8) |  |
| ≥30 | 134 | (30.2) | 38 | (26.2) |  |
| **Medications** |  |  |  |  |  |
| Oral glucose-lowering drugs | 428 | (96.4) | 139 | (95.9) | 0.966 |
| Insulin | 113 | (25.5) | 48 | (33.1) | 0.091 |
| Blood-pressure lowering drugs | 349 | (78.6) | 112 | (77.2) | 0.819 |
| Lipid lowering drugs | 364 | (82.0) | 116 | (80.0) | 0.681 |
| **History of complications** |  |  |  |  |  |
| Coronary heart disease | 21 | (4.7) | 9 | (6.2) | 0.628 |
| Peripheral vascular disease | 3 | (0.7) | 1 | (0.7) | 1 |
| Stroke | 7 | (1.6) | 2 | (1.4) | 1 |
| Heart failure hospitalization | 2 | (0.5) | 3 | (2.1) | 0.186 |
| Chronic kidney disease | 27 | (6.1) | 9 | (6.2) | 1 |
| Any-site cancer | 15 | (3.4) | 10 | (6.9) | 0.112 |
| **Lifestyle factor** |  |  |  |  |  |
| Adherence to a balanced diet in past 3 months | 419 | (94.4) | 141 | (97.2) | 0.243 |
| Frequency of exercise per week |  |  |  |  |  |
| No regular activity | 142 | (32.0) | 44 | (30.3) | 0.002 |
| Less than 3 times | 177 | (39.9) | 43 | (29.7) |  |
| 3 to 4 times | 43 | (9.7) | 30 | (20.7) |  |
| 5 times or more | 82 | (18.5) | 27 | (18.6) |  |
| Missing | 0 | (0.0) | 1 | (0.7) |  |
| **Smoking** |  |  |  |  |  |
| Current | 92 | (20.7) | 30 | (20.7) | 0.398 |
| Ex-smoker | 57 | (12.8) | 25 | (17.2) |  |
| Never | 295 | (66.4) | 90 | (62.1) |  |
| **Use of alcohol** |  |  |  |  |  |
| Regular | 14 | (3.2) | 1 | (0.7) | 0.004 |
| Occasional | 178 | (40.1) | 38 | (26.2) |  |
| Ex-drinker | 19 | (4.3) | 7 | (4.8) |  |
| Never | 233 | (52.5) | 99 | (68.3) |  |
| **Taking care of children (<18 years old)** | 147 | (33.1) | 65 | (44.8) | 0.014 |
| ***Work-related factors*** |  |  |  |  |  |
| **Employment status** |  |  |  |  |  |
| Full time | 366 | (82.4) | 42 | (29.0) | <0.001 |
| Part time | 24 | (5.4) | 14 | (9.7) |  |
| Self-employed | 54 | (12.2) | 10 | (6.9) |  |
| Not working | 0 | (0.0) | 77 | (53.1) |  |
| Missing | 0 | (0.0) | 2 | (1.4) |  |
| **Work schedule** |  |  |  |  |  |
| Regular working hours | 363 | (81.8) | 39 | (26.9) | <0.001 |
| Shift work / self-employment | 81 | (18.2) | 24 | (16.6) |  |
| Not working | 0 | (0.0) | 76 | (52.4) |  |
| Missing | 0 | (0.0) | 6 | (4.1) |  |
| **Sick leave days** | 0.48 | (1.48) | 1.12 | (11.23) | 0.236 |
| **Annual salary (USD$)** |  |  |  |  |  |
| <22,000 | 45 | (10.1) | 25 | (17.2) | <0.001 |
| ≥22,000 <29,000 | 69 | (15.5) | 19 | (13.1) |  |
| ≥29,000 <45,000 | 171 | (38.5) | 19 | (13.1) |  |
| ≥45,000 <71,000 | 95 | (21.4) | 14 | (9.7) |  |
| ≥71,000 | 64 | (14.4) | 9 | (6.2) |  |
| Missing | 0 | (0.0) | 59 | (40.7) |  |

# Supplementary table 3. Patient characteristics stratified by sex

|  | **Male**  (n=266)  N (%) /  Mean (SD) | | **Female**  (n=178)  N (%) /  Mean (SD) | | **P-value** |
| --- | --- | --- | --- | --- | --- |
|  |  |  |  |  |  |
|  |  |  |  |  |  |
| ***Personal Characteristics*** |  |  |  |  |  |
| **Education** |  |  |  |  |  |
| College or above | 109 | (41.0) | 65 | (36.5) | 0.608 |
| High school | 67 | (25.2) | 46 | (25.8) |  |
| Middle, primary school or lower | 90 | (33.8) | 67 | (37.6) |  |
| **Age** |  |  |  |  |  |
| < 40 years old | 64 | (24.1) | 42 | (23.6) | 0.963 |
| ≥ 40 < 50 years old | 151 | (56.8) | 100 | (56.2) |  |
| ≥ 50 years old | 51 | (19.2) | 36 | (20.2) |  |
| **Duration of diabetes** |  |  |  |  |  |
| <5 years | 45 | (16.9) | 21 | (11.8) | 0.014 |
| ≥ 5 < 10 years old | 95 | (35.7) | 51 | (28.7) |  |
| ≥ 10 < 15 years old | 77 | (28.9) | 51 | (28.7) |  |
| ≥ 15 years old | 49 | (18.4) | 55 | (30.9) |  |
| ***Clinical characteristics*** |  |  |  |  |  |
| **HbA1c (%)** |  |  |  |  |  |
| <7.0 | 149 | (56.0) | 87 | (48.9) | 0.168 |
| ≥7.0 | 117 | (44.0) | 91 | (51.1) |  |
| **Low-Density Lipoprotein Cholesterol (mmol/L)** |  |  |  |  |  |
| <2.60 | 204 | (76.7) | 130 | (73.0) | 0.086 |
| ≥2.60 <3.35 | 38 | (14.3) | 38 | (21.3) |  |
| ≥3.35 | 24 | (9.0) | 10 | (5.6) |  |
| **Albuminuria status** |  |  |  |  |  |
| Yes (uACR ≥3 mg/mmol / ≥30 mg/g) | 29 | (10.9) | 18 | (10.1) | 0.914 |
| **Estimated glomerular filtration rate (eGFR) (mL/min/1.73m^2^)** |  |  |  |  |  |
| <60 | 17 | (6.4) | 10 | (5.6) | 0.002 |
| ≥60 <90 | 67 | (25.2) | 21 | (11.8) |  |
| ≥90 | 182 | (68.4) | 147 | (82.6) |  |
| **Body Mass Index (kg/m^2^)** |  |  |  |  |  |
| <25 | 72 | (27.1) | 50 | (28.1) | 0.959 |
| ≥25 <30 | 114 | (42.9) | 74 | (41.6) |  |
| ≥30 | 80 | (30.1) | 54 | (30.3) |  |
| **Medications** |  |  |  |  |  |
| Oral glucose-lowering drugs | 254 | (95.5) | 174 | (97.8) | 0.32 |
| Insulin | 58 | (21.8) | 55 | (30.9) | 0.041 |
| Blood-pressure lowering drugs | 222 | (83.5) | 127 | (71.3) | 0.003 |
| Lipid lowering drugs | 232 | (87.2) | 132 | (74.2) | 0.001 |
| **History of complications** |  |  |  |  |  |
| Coronary heart disease | 15 | (5.6) | 6 | (3.4) | 0.381 |
| Peripheral vascular disease | 1 | (0.4) | 2 | (1.1) | 0.725 |
| Stroke | 4 | (1.5) | 3 | (1.7) | 1 |
| Heart failure hospitalization | 2 | (0.8) | 0 | (0.0) | 0.663 |
| Chronic kidney disease | 16 | (6.0) | 11 | (6.2) | 1 |
| All-site cancer | 9 | (3.4) | 6 | (3.4) |  |
| **Lifestyle factor** |  |  |  |  |  |
| Adherence to a balanced diet in past 3 months | 251 | (94.4) | 168 | (94.4) | 1 |
| Frequency of exercise per week |  |  |  |  |  |
| No regular activity | 78 | (29.3) | 64 | (36.0) | 0.239 |
| Less than 3 times | 108 | (40.6) | 69 | (38.8) |  |
| 3 to 4 times | 31 | (11.7) | 12 | (6.7) |  |
| 5 times or more | 49 | (18.4) | 33 | (18.5) |  |
| **Smoking** |  |  |  |  |  |
| Current | 76 | (28.6) | 16 | (9.0) | <0.001 |
| Ex-smoker | 41 | (15.4) | 16 | (9.0) |  |
| Never | 149 | (56.0) | 146 | (82.0) |  |
| **Use of alcohol** |  |  |  |  |  |
| Regular | 12 | (4.5) | 2 | (1.1) | <0.001 |
| Occasional | 124 | (46.6) | 54 | (30.3) |  |
| Ex-drinker | 15 | (5.6) | 4 | (2.2) |  |
| Never | 115 | (43.2) | 118 | (66.3) |  |
| **Taking care of children (<18 years old)** | 132 | (49.6) | 56 | (33.1) | 0.001 |
| ***Work-related factors*** |  |  |  |  |  |
| **Employment status** |  |  |  |  |  |
| Full time | 211 | (79.3) | 155 | (87.1) | <0.001 |
| Part time | 4 | (1.5) | 20 | (11.2) |  |
| Self-employed | 51 | (19.2) | 3 | (1.7) |  |
| **Work schedule** |  |  |  |  |  |
| Regular working hours | 212 | (79.7) | 151 | (84.8) | 0.212 |
| Shift work / self-employment | 54 | (20.3) | 27 | (15.2) |  |
| **Sick leave days** | 0.45 | (1.66) | 0.52 | (1.18) | 0.643 |
| **Annual salary (USD$)** |  |  |  |  |  |
| <22,000 | 9 | (3.4) | 36 | (20.2) | <0.001 |
| ≥22,000 <29,000 | 34 | (12.8) | 35 | (19.7) |  |
| ≥29,000 <45,000 | 114 | (42.9) | 57 | (32.0) |  |
| ≥45,000 <71,000 | 62 | (23.3) | 33 | (18.5) |  |
| ≥71,000 | 47 | (17.7) | 17 | (9.6) |  |
